# The Preschool-Aged and School-Aged Children Present Different Odds of Mortality than Adults in Southern Taiwan: A Cross-Sectional Retrospective Analysis

**DOI:** 10.3390/ijerph15050858

**Published:** 2018-04-25

**Authors:** Shu-Hui Peng, Chun-Ying Huang, Shiun-Yuan Hsu, Li-Hui Yang, Ching-Hua Hsieh

**Affiliations:** 1 Department of Plastic Surgery, Kaohsiung Chang Gung Memorial Hospital, Chang Gung University College of Medicine, Kaohsiung 83301, Taiwan; pshui@cgmh.org.tw; 2 Department of Trauma Surgery, Kaohsiung Chang Gung Memorial Hospital, Chang Gung University College of Medicine, Kaohsiung 83301, Taiwan; junyinhaung@yahoo.com.tw (C.-Y.H.); ah.lucy@hotmail.com (S.-Y.H.); 3 Department of Nursing, Kaohsiung Chang Gung Memorial Hospital, Chang Gung University College of Medicine, Kaohsiung 83301, Taiwan; 4 Department of Nursing, Meiho University, Pingtung County 91202, Taiwan

**Keywords:** preschool-aged children, school-aged children, injury, pediatric trauma, mortality, trauma registry

## Abstract

*Background*: This study aimed to profile the epidemiology of injury among preschool-aged and school-aged children in comparison to those in adults. *Methods*: According to the Trauma Registry System of a level I trauma center, the medical data were retrieved from 938 preschool-aged children (aged less than seven years), 670 school-aged children (aged 7–12 years), and 16,800 adults (aged 20–64 years) between 1 January 2009 and 31 December 2016. Two-sided Pearson’s, chi-squared, and Fisher’s exact tests were used to compare categorical data. A one-way analysis of variance (ANOVA) with the Games-Howell post-hoc test was used to assess the differences in continuous variables among different groups of patients. The mortality outcomes of different subgroups were assessed by a multivariable regression model under the adjustment of sex, injury mechanisms, and injury severity. *Results*: InFsupppjury mechanisms in preschool-aged and school-aged children were remarkably different from that in adults; in preschool-aged children, burns were the most common cause of injury requiring hospitalization (37.4%), followed by falls (35.1%) and being struck by/against objects (11.6%). In school-aged children, injuries were most commonly sustained from falls (47.8%), followed by bicycle accidents (14%) and being struck by/against objects (12.5%). Compared to adults, there was no significant difference of the adjusted mortality of the preschool-aged children (AOR = 0.9; 95% CI 0.38–2.12; *p* = 0.792) but there were lower adjusted odds of mortality of the school-aged children (AOR = 0.4; 95% CI 0.10–0.85; *p* = 0.039). The school-aged children had lower odds of mortality than adults (OR, 0.2; 95% CI, 0.06–0.74; *p* = 0.012), but such lower odds of risk of mortality were not found in preschool-aged children (OR, 0.7; 95% CI, 0.29–1.81; *p* = 0.646). *Conclusions*: This study suggests that specific types of injuries from different injury mechanisms are predominant among preschool-aged and school-aged children. The school-aged children had lower odds of mortality than adults; nonetheless there was no difference in mortality rates of preschool-aged children than adults, with or without controlling for sex, injury mechanisms and ISS. These results highlight the importance of injury prevention, particularly for preschool-aged children in Southern Taiwan.

## 1. Introduction

This study showed that one in four children in the United States will sustain an unintentional injury requiring emergency treatment [1,2], leading to over 8.7 million hospital visits each year [3,4]. In addition, trauma is the leading cause of mortality and acquired disability in children [5,6,7], causing 12,000 injury-related deaths [8] and accounting for 34% of all child mortality annually in the United States [9]. However, compared to adults, children had lower reported trauma-related mortality rates in the United States: The fatalities were 12.69, 3.69 and 3.7 per 100,000 persons in pediatric population aged 0–4 years, 5–9 years and 10–14 years respectively; compared to 45.30 fatalities per 100,000 persons in adults aged 20–64 years [9].

Compared with adults, children present different patterns of injury [10]. The pattern and circumstances of injuries change as children progress with age [11]. Obviously, the etiology of injuries is complex; it differs from children to adults and even from country to country. Furthermore, the physiological responses [12,13] and capacity to thrive [14] might be different in children and adults. Therefore, to reduce the injury to children, it is important to understand the associated injury patterns and outcomes for children [15]. However, in Taiwan, most of previous studies on children injury focus on trauma due to specific etiology such as child abuse [16,17], burns [18] or head injuries [16,19]. Therefore, to explore the epidemiological profile of child injury under the hypothesis that the children would have a lower mortality rate than adults in Taiwan, the first objective of this study was to systematically examine the rates of child injury among preschool-aged and school-aged children compared to those of adults. The secondary objective was to investigate whether preschool-aged and school-aged children had a better expected mortality outcome than adults, in the absence or presence of adjusting potential confounders including sex, injury mechanism, and injury severity. This article focused on trauma of children and did not include the teenagers, who are at high risk for experiencing traumatic events and respond differently to injury [20]. In this goal, the mortality rate would be measured as the primary outcome.

## 2. Methods

### 2.1. Ethical Statement

The retrospective study was performed after receiving approval from the institutional review board (IRB) of Chang Gung Memorial Hospital (approval number: 201701671B0). Informed consent requirements were waived according to the regulations of IRB. All analyses were conducted using de-identified secondary data, with no means to link the information to individual respondents.

### 2.2. Study Design

**Figure 1:**
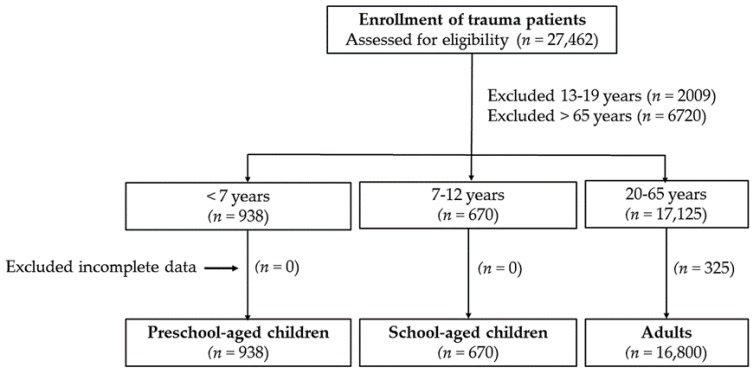
Flow chart of the distribution of the studied population into preschool-aged children, school-aged children, and adults.

In this study, patients were stratified as preschool-aged children (aged less than 7 years), school-aged children (aged 7–12 years), and adults (aged 20–64 years). Out of the 27,462 hospitalized patients enrolled in the Trauma Registry System from 1 January 2009 to 31 December 2016 for all-causes of trauma, 938 were preschool-aged children, 670 were school-aged children, and 16,800 were adults; the 325 adult patients who had incomplete registered data were excluded (Figure 1). We retrieved the detailed patient information from the registry database, including age, trauma mechanism, helmet use, initial Glasgow Coma Scale (GCS) score upon arrival at the emergency department, Injury Severity Score (ISS), Abbreviated Injury Scale (AIS) score in each body region, rates of sustained injuries in each body region, and in-hospital mortality [21,22]. The AIS is an anatomically based measurement of injury severity to rank specific injuries as minor (1), moderate (2), serious (3), severe (4), critical (5), and unsurvivable (6). The ISS was created based on the AIS severity values using the sum of the squares of the severity digit in the AIS of most severe injuries in three of six body regions [23].

### 2.3. Statistical Analysis

The ISS is expressed as the median and interquartile range (IQR, Q1–Q3). The obtained data were compared using the IBM SPSS Statistics for Windows version 22.0 (IBM Corp., Armonk, NY, USA). Categorical data were compared using either a chi-squared test or a two-sided Fisher’s exact test. The odds ratios (ORs) of the associated conditions and bone fractures of the patients were calculated with 95% confidence intervals (CIs). The homogeneity of variance of the continuous data was initially estimated using Levene’s test, then a one-way analysis of variance (ANOVA) with the Games-Howell post-hoc test was used to assess the differences of continuous variables among different groups of patients. The continuous data were expressed as mean ± standard deviation. The difference in ISS distribution among different subgroups of patients was analyzed using Kruskal-Wallis nonparametric test. The ISS was expressed as the median and interquartile range (IQR, Q1–Q3). The adjusted odds ratios (AORs) and 95% CIs for mortality were calculated by using a multivariable regression model adjusted for sex, trauma mechanisms, and ISS. The mortality outcomes were assessed by a binary logistic regression. The mortality outcomes in different subgroups were assessed with a multivariable regression model adjusted for sex, injury mechanisms, and injury severity. *p*-values < 0.05 were considered statistically significant.

## 3. Results

### 3.1. Injury Characteristics and Severity of the Patients

As shown in Table 1, there was no significant difference in sex predominance between preschool-aged, school-aged children and adults. The injury mechanisms in preschool-aged and school-aged children were different from those in adults; in preschool-aged children, burns were the most common cause of injury requiring hospitalization (37.4%), followed by falls (35.1%) and being struck by/against an object (11.6%). In school-aged children, injuries were most commonly sustained from falls, followed by bicycle accidents (14%) and being struck by/against an object (12.5%). Motorcycle accidents occurred more frequently in adults and comprised around half of the admitted patients. Injuries such as injuries from motorcycle pillion accidents, injuries due to falls, and burn injuries were significantly higher in both preschool-aged and school-aged children than adult patients. The helmet use was significantly associated with a lower odds of head injury, defined by head AIS ≥2, in school-aged children (OR 0.1; 95% CI 0.02–0.37; *p* < 0.001) but not in preschool-aged children (OR 0.7; 95% CI 0.21–2.31; *p* = 0.768) (Supplementary Materials Table S1). The percentage of preschool-aged children, but no school-aged children, sustaining pedestrian and bite-related injuries is higher than that of adults (Table 1).

Most of the patients had a GCS score of ≥13 and fewer had a GCS score of ≤8. The GCS scores in preschool-aged and school-aged children were significantly lower than those of adults, notwithstanding that the GCS score difference between these groups was less than one point (Table 2). The AIS analysis revealed that preschool-aged and school-aged children had lower rates of injuries in each body region than adults; however, preschool-aged children had higher rates of external injuries, predominantly burns, than adults. Moreover, preschool-aged and school-aged children had a significantly lower ISS compared with adults (Table 2). When stratified using ISS (<16, 16–24 or ≥25), fewer preschool-aged and school-aged children had an ISS of ≥25 and 16–24, respectively, and more had an ISS of <16 than adults (Table 1).

As regards associated injuries, preschool-aged and school-aged children had lower odds of different injuries in the six main body regions (Supplementary Materials Tables S2 and S3). On the other hand, there was a 4.4-fold (95% CI, 3.70–5.25; *p* < 0.001) and 4.1-fold (95% CI, 3.35–5.07; *p* < 0.001) increase in the odds of humeral fracture among preschool-aged and school-aged children respectively (Table 3). Compared with adults, preschool-aged children had a lower odds of radial fracture (OR, 0.4; 95% CI, 0.30–0.55; *p* < 0.001) and ulnar fracture (OR, 0.7; 95% CI, 0.47–0.96; *p* = 0.032), whereas school-aged children had a higher odds of radial fracture (OR, 3.2; 95% CI, 2.68–3.81; *p* < 0.001) and ulnar fracture (OR, 3.2; 95% CI, 2.59–4.05; *p* < 0.001).

### 3.2. Patient Outcomes and Associated Injuries

As shown in Table 4, the mortality rates between preschool-aged children and adults were not significantly different. By contrast, the mortality odds ratio in school-aged children was lower than that in adults (OR, 0.3; 95% CI, 0.09–0.92; *p* = 0.031). Further, compared to adults, there was no significant difference in the adjusted mortality of the preschool-aged children (AOR = 0.9; 95% CI 0.38–2.12; *p* = 0.792) but lower adjusted odds of mortality of the school-aged children (AOR = 0.4; 95% CI 0.10–0.85; *p* = 0.039). Notably, in this study, although there was a higher occurrence of burn injuries in preschool-aged children (37.4%, *n* = 351) than school-aged children (11.3%, *n* = 76) and adults (3.4%, *n* = 570), the mortality of preschool-aged children (0.6%, *n* = 2) with a burn was significantly lower (OR = 0.3; 95% CI 0.05–1.01; *p* = 0.037) than that of adults (2.5%, *n* = 14) and the mortality rates of school-aged children (3.9%, *n* = 3) and adults were not significantly different (OR = 1.6; 95% CI 0.46–5.82; *p* = 0.708). Therefore, the unexpectedly high mortality rates of preschool-aged children cannot be explained by the higher proportion of burn injuries in preschool-aged patients.

## 4. Discussion

Findings of this study are in accordance with literature that suggests there are certain types of injuries that are common in specific child age group. In a study on 2.4 million pediatric traumatic injury cases in the US from 2000–2011, motorcycle accidents were the most common mechanism of traumatic injuries for all age groups [24]. In children less than 5 years old, burns and abuse were the second and third most common causes of severe traumatic injury. In children aged 5–9 years, falls and animal bites were the second and third most common causes of injuries. Sports were the second most common cause of injury in children aged 10–14 years [24]. This study also found the changing patterns of injuries with age. Burns were the most common injury in preschool-aged children, followed by falls and being struck by/against objects. In school-aged children, falls were the most commonly sustained injury, followed by bicycle accidents, and being struck by/against objects. In addition, injuries as a motorcycle pillion, injuries due to falls, and burn injuries were significantly higher in both preschool-aged and school-aged children than in adult patients. The percentage of preschool-aged children sustaining pedestrian and bite-related injuries is significantly higher than that of adults, but for school-aged children the percentage is not significantly higher than that of adults. Furthermore, bicycle injuries were significantly higher, particularly in school-aged children.

Regarding the associated injuries, this study revealed that preschool-aged and school-aged children had higher odds of humeral fracture than adults. In addition, school-aged children had higher odds of radial fracture and ulnar fracture. The incidence of fractures in children is two times higher than that in adults [25]. From birth until the age of 12 years, all major series have demonstrated a linear increase in the incidence of fractures by age [25,26]. The difference in the rates of fractures occurs at a certain age when behaviors change, and the boys tend to develop a more aggressive and risk-taking behavior [25]. In this study, the incidence of fractures in the upper limbs were three-folds higher than that in the lower limbs. This finding is higher than that reported in a previous study conducted in a Taiwanese cohort, which reported that the rates of upper limb injuries were two times higher than those of lower limb injuries [27]. Upper extremity fractures were prevalent among children where shock was absorbed in the upper limb after falling from a climbing equipment or slide [28,29]. Rennie et al. had reported that fracture of the distal radius (15.3%) are the most frequent single fractures that occur among children, followed by finger fractures (14%), radial and ulnar fractures (8%), distal humerus fractures (7.2%), and clavicle fractures (6.8%) [2].

Previous studies reported that mortality in the pediatric population in all age groups were significantly lower than that in adults [9]. In this study, the odds of mortality in school-aged children was lower than that in adults, but there was no difference in mortality rates between preschool-aged children and adults, either in the presence or absence of controlling for sex, injury mechanisms, and ISS. However, the reason behind the failure to reduction in the odds of mortality among preschool-aged children remains unclear and should raise alarm. In this study, the incidence of other commonly sustained injuries with an associated mortality, including hemothorax or pneumothorax, hepatic injury, and pelvic fracture, was significantly lower in preschool-aged child than in adults. In particular, the incidence of TBI, including neurologic deficit, subdural hemorrhage, subarachnoid hemorrhage, intracerebral hematoma and cerebral contusion in preschool-aged children was even lower than that in adults.

Although majority of childhood head injuries are minor, approximately 5% of these injuries can cause mortality or lead to intracranial complications [30]. In a study conducted in 216 participating hospitals, intracranial injury or skull fracture occurred in one out of ten children, with the overall mortality being 0.4%, and these injuries were predominant among victims of motor vehicle accidents or abusive head trauma [31]. Unlike those studies conducted in Western countries, the occurrence of motor vehicle accidents were much lower in Taiwan. In this study, only 0.7% and 1.2% of preschool-aged and school-age child respectively had sustained injuries as a passenger in the motor vehicle accidents. In Taiwan, nearly 60% of all trauma injuries and driving fatalities involving motorcycle accidents [32]. It had been reported that, in Taiwan, the mortality rate of motorcyclists using helmets was significantly lower than those not using helmets (1.1% vs. 4.2%, respectively; OR, 0.2; 95% CI, 0.17–0.37; *p* < 0.001) [33]. However, in this study, the rates of injuries as a motorcycle pillion in both preschool-aged and school-aged children were higher than that in adult patients. Besides, helmet use was significantly associated with lower odds of head injury in school-aged children (OR 0.1; 95% CI 0.02–0.37; *p* < 0.001) but not in preschool-aged children (OR 0.7; 95% CI 0.21–2.31; *p* = 0.768). Whether the failure to protect a fatal head injury in preschool-aged child is attributable to a mismatch between the sizes of helmets and their heads requires further investigation to confirm. However, the implementation of more effective methods to reduce motorcycle accidents and the strict use of a protective helmets in motorcycle pillions in accordance with the traffic laws may be effective to reduce the occurrence of head injury and its associated mortality.

This study also revealed that preschool-aged children are at heightened risk for pedestrian injuries than adults. Interventions such as modification of the road environment [34], implementation of a higher number of student crossings, a wider road width, the presence of crosswalks, student-friendly facilities at the intersection, and four-way intersections [35] may help to reduce the pedestrian crashes. For example, the implementation of the Safe Routes to School (SRTS) program in New York City has contributed to a substantial 44% reduction in pedestrian injuries among school-aged children [36].

This study had several limitations. First, although the cutoff value for preschool-aged vs. school-aged children was chosen based on the situation in Taiwan, the profile of childhood injury may change greatly over the first three or four years of life. Therefore, grouping the first seven years together for analysis may have lead to a selection bias. Second, an inherent selection bias already existed because of the retrospective study design, particularly when considering the circumstances of injuries, the impact force of each injury, and the pre-existing comorbidities that were left unrecorded (although the incidence of these comorbidities is expected to be much lower in children than that in adults). Third, this study focused on hospitalized pediatric patients only; however, many minor injuries sustained at certain sites are manageable and do not require hospital admissions, which may lead to underestimation on the incidence of associated illness, particularly the fractures, burns, or dog bite injuries, and may result in a selection bias. Fourth, these data systems do not capture important non-fatal outcomes, such as functional status or quality of life outcomes. Considering the difference of the remaining lifespan in the pediatric and adult victims, it is important to evaluate the burden of trauma in the lives of pediatric patients. Fifth, the number of preschool-aged and school-aged children with fatal injuries was lower than that of adults, which may result in a potential source of methodological bias in the assessment. Finally, the study was conducted only in patients admitted in a level I trauma center; whether children received suboptimal prehospital care compared to the adults at a level I trauma center and not at a pediatric trauma center would raise further debate; so the results of this study remained inconclusive [37,38,39].

## 5. Conclusions

This study suggests that preschool-aged and school-aged children dominate specific types of injuries from different injury mechanisms. The school-aged children had lower odds of mortality than adults. However, such reduction in mortality was not seen in the preschool-aged children compared to adults, either in the presence or absence of controlling for sex, injury mechanisms, and ISS. These results highlighted the importance of injury prevention particularly in the preschool-aged children in Southern Taiwan.

## Figures and Tables

**Table 1:** Injury characteristics of categorical variables in preschool-aged children, school-aged children, and adults.

Variables	Preschool-Aged Child *n* = 938		School-Aged Child *n* = 670		Adult *n* = 16,800		*P* Preschool-Aged Child vs. Adult	*P* Preschool-Aged Child vs. Adult
Sex, n (%)							0.084	0.351
Male	554	(59.1)	427	(63.7)	10,399	(61.9)		
Female	384	(40.9)	243	(36.3)	6401	(38.1)		
Mechanisms, n (%)								
Driver (motor vehicle)	0	(0.0)	0	(0.0)	328	(2.0)	<0.001	<0.001
Passenger (motor vehicle)	7	(0.7)	8	(1.2)	166	(1.0)	0.505	0.690
Driver (motorcycle)	0	(0.0)	3	(0.4)	8012	(47.7)	<0.001	<0.001
Pillion (motorcycle)	63	(6.7)	64	(9.6)	358	(2.1)	<0.001	<0.001
Bicyclist	28	(3.0)	94	(14.0)	435	(2.6)	0.460	<0.001
Pedestrian	36	(3.8)	16	(2.4)	252	(1.5)	<0.001	0.076
Fall	329	(35.1)	320	(47.8)	3388	(20.2)	<0.001	<0.001
Struck by/against	109	(11.6)	84	(12.5)	3202	(19.1)	<0.001	<0.001
Bite	15	(1.6)	5	(0.7)	89	(0.5)	<0.001	0.588
Burn	351	(37.4)	76	(11.3)	570	(3.4)	<0.001	<0.001
GCS, n (%)								
≤8	19	(2.0)	10	(1.5)	722	(4.3)	0.001	<0.001
9–12	12	(1.3)	12	(1.8)	523	(3.1)	0.002	0.051
≥13	907	(96.7)	648	(96.7)	15,555	(92.6)	<0.001	<0.001
AIS n (%)								
Head/Neck	156	(16.6)	95	(14.2)	3870	(23.0)	<0.001	<0.001
Face	76	(8.1)	69	(10.3)	2926	(17.4)	<0.001	<0.001
Thorax	12	(1.3)	8	(1.2)	2160	(12.9)	<0.001	<0.001
Abdomen	30	(3.2)	26	(3.9)	1104	(6.6)	<0.001	0.006
Extremity	390	(41.6)	453	(67.6)	12,043	(71.7)	<0.001	0.023
External	387	(41.3)	97	(14.5)	2454	(14.6)	<0.001	0.956
ISS								
<16	875	(93.3)	623	(93.0)	14,440	(86.0)	<0.001	<0.001
16–24	46	(4.9)	40	(6.0)	1598	(9.5)	<0.001	0.002
≥25	17	(1.8)	7	(1.0)	762	(4.5)	<0.001	<0.001

AIS = Abbreviated Injury Scale; CI = Confidence Interval; GCS = Glasgow Coma Scale; ISS = injury severity score; IQR = Interquartile Range.

**Table 2:** Injury characteristics and outcomes of continuous variables in preschool-aged children, school-aged children, and adults.

	**Preschool-Aged Child *n* = 938**	**School-Aged Child *n* = 670**	**Adult *n* = 16,800**	***ANOVA P***	**Comparison between**		**Mean Difference**	**Post-hoc *P***
GCS	14.73 ± 1.46	14.76 ± 1.29	14.40 ± 2.10	<0.001	Preschool-aged child	School-aged child	−0.03	0.925
						adult	0.33	<0.001
					School-aged child	Adult	0.36	<0.001
	**Preschool-Aged Child *n* = 938**	**school-Aged Child *n* = 670**	**Adult *n* = 16,800**	**Kruskal-Wallis *P***			**Median Difference**	**Post-hoc *P***
ISS	4 (1–4)	4 (4–9)	5 (4–9)	<0.001	Preschool-aged child	School-aged child	0	<0.001
						Adult	−1	<0.001
					School-aged child	Adult	−1	<0.001

GCS = Glasgow coma scale; ISS = injury severity score.

**Table 3:** Associated injuries with higher odds of incidence among preschool-aged and school-aged children than in adults.

Variables	Preschool-Aged Child		School-Aged Child		Adult		OR (95% CI)		*P*	OR (95% CI)		*P*
	*n* = 938		*n* = 670		*n* = 16,800		Preschool-Aged Child vs. Adult			School-Aged Child vs. Adult		
Humeral fracture	182	(19.4)	123	(18.4)	870	(5.2)	4.4	(3.70–5.25)	<0.001	4.1	(3.35–5.07)	<0.001
Radial fracture	44	(4.7)	187	(27.9)	1814	(10.8)	0.4	(0.30–0.55)	<0.001	3.2	(2.68–3.81)	<0.001
Ulnar fracture	33	(3.5)	100	(14.9)	864	(5.1)	0.7	(0.47–0.96)	0.032	3.2	(2.59–4.05)	<0.001

**Table 4:** Outcomes of categorical variables in preschool-aged children, school-aged children, and adults.

Variables	Preschool-Aged Child *n* = 938		School-Aged Child *n* = 670		Adult *n* = 16,800		OR (95% CI) *P* Preschool-Aged Child vs. Adult			OR (95% CI) *P* School-Aged Child vs. Adult		
Mortality, n (%)	8	(0.9)	3	(0.4)	254	(1.5)	0.6	(0.28–1.14)	0.124	0.3	(0.09–0.92)	0.031
Adjusted mortality							0.9	(0.38–2.12)	0.792	0.4	(0.10–0.85)	0.039

CI = Confidence Interval; IQR = Interquartile Range; OR = odds ratio.

## Supplementary Materials

The following are available online at http://www.mdpi.com/1660-4601/15/5/858/s1. Table S1: The association of head injury and helmet use in the motorcycle pillion of preschool-aged and school-aged children; Table S2: Associated injuries in the six main body regions; Table S3: Comparison of associated injuries in the six main body regions.
