# Supplementary material for: The Preschool-Aged and School-Aged Children Present Different Odds of Mortality than Adults in Southern Taiwan: A Cross-Sectional Retrospective Analysis
Source: Int J Environ Res Public Health. 2018 Apr 25;15(5):858. doi: 10.3390/ijerph15050858 (PMC5981897; doi:10.3390/ijerph15050858)
Supplement: Supplementary file 1 [file ijerph-15-00858-s001.pdf]

Table S1. The association of head injury and helmet use in the motorcycle pillion of preschool-aged and school-aged children.

Preschool-aged child	Helmet(+) n=18	Helmet(-) n=45	OR (95%CI)	*P*
				0.768
Head injury (Yes)	5 (27.8)	16 (35.6)	0.7 (0.21-2.31)	
Head injury (No)	13 (72.2)	29 (64.4)	1.4 (0.43-4.76)	

school-aged child	Helmet(+) n=35	Helmet(-) n=29	OR (95%CI)	*P*
				<0.001
Head injury (Yes)	2 (5.7)	13 (44.8)	0.1 (0.02-0.37)	
Head injury (No)	33 (94.3)	16 (55.2)	13.4 (2.70-66.67)	

Table S2. Associated injuries in the six main body regions.

Variables	preschool-aged child n=938	school-aged child n=670	adult n=16,800
**Head trauma, n (%)**			
**Neurologic deficit**	1 (0.1)	2 (0.3)	171 (1.0)
**Cranial fracture**	61 (6.5)	31 (4.6)	941 (5.6)
**Epidural hematoma**	32 (3.4)	31 (4.6)	571 (3.4)
**Subdural hematoma**	47 (5.0)	31 (4.6)	1274 (7.6)
**Subarachnoid hemorrhage**	32 (3.4)	27 (4.0)	1323 (7.9)
**Intracerebral hematoma**	5 (0.5)	10 (1.5)	307 (1.8)
**Cerebral contusion**	20 (2.1)	7 (1.0)	623 (3.7)
**Cervical vertebral fracture**	1 (0.1)	1 (0.1)	182 (1.1)
**Maxillofacial trauma, n (%)**			
**Orbital fracture**	4 (0.4)	6 (0.9)	378 (2.3)
**Nasal fracture**	2 (0.2)	3 (0.4)	210 (1.3)
**Maxillary fracture**	5 (0.5)	9 (1.3)	1094 (6.5)
**Mandibular fracture**	4 (0.4)	9 (1.3)	414 (2.5)
**Thoracic trauma, n (%)**			
**Rib fracture**	2 (0.2)	2 (0.3)	1578 (9.4)
**Sternal fracture**	0 (0.0)	0 (0.0)	26 (0.2)
**Hemothorax**	0 (0.0)	0 (0.0)	259 (1.5)
**Pneumothorax**	4 (0.4)	2 (0.3)	301 (1.8)
**Hemopneumothorax**	2 (0.2)	1 (0.1)	229 (1.4)
**Lung contusion**	8 (0.9)	0 (0.0)	220 (1.3)
**Thoracic vertebral fracture**	0 (0.0)	0 (0.0)	164 (1.0)
**Abdominal trauma, n (%)**			
**Intra-abdominal injury**	15 (1.6)	8 (1.2)	276 (1.6)
**Hepatic injury**	17 (1.8)	4 (0.6)	344 (2.0)
**Splenic injury**	8 (0.9)	7 (1.0)	177 (1.1)
**Retroperitoneal injury**	0 (0.0)	0 (0.0)	44 (0.3)
**Renal injury**	2 (0.2)	5 (0.7)	91 (0.5)
**Urinary bladder injury**	2 (0.2)	0 (0.0)	24 (0.1)

**Lumbar vertebral fracture**	0 (0.0)	0 (0.0)	311 (1.9)
**Sacral vertebral fracture**	0 (0.0)	0 (0.0)	113 (0.7)
**Extremity trauma, n (%)**			
**Scapular fracture**	4 (0.4)	0 (0.0)	373 (2.2)
**Clavicle fracture**	7 (0.7)	7 (1.0)	1513 (9.0)
**Humeral fracture**	182 (19.4)	123 (18.4)	870 (5.2)
**Radial fracture**	44 (4.7)	187 (27.9)	1814 (10.8)
**Ulnar fracture**	33 (3.5)	100 (14.9)	864 (5.1)
**Metacarpal fracture**	4 (0.4)	3 (0.4)	506 (3.0)
**Pelvic fracture**	6 (0.6)	2 (0.3)	497 (3.0)
**Femoral fracture**	41 (4.4)	35 (5.2)	1417 (8.4)
**Patella fracture**	0 (0.0)	1 (0.1)	463 (2.8)
**Tibial fracture**	17 (1.8)	22 (3.3)	952 (5.7)
**Fibular fracture**	19 (2.0)	14 (2.1)	702 (4.2)
**Calcaneal fracture**	0 (0.0)	5 (0.7)	906 (5.4)
Metatarsal fracture	2 (0.2)	5 (0.7)	560 (3.3)

Table S3. Comparison of associated injuries in the six main body regions.

	**preschool-aged child vs. adult**		**school-aged child vs. adult**	
Variables	**OR (95% CI)**	***P***	**OR (95% CI)**	***P***
**Head trauma, n (%)**				
**Neurologic deficit**	0.1 (0.02-0.74)	0.005	0.3 (0.07-1.18)	0.071
**Cranial fracture**	1.2 (0.90-1.53)	0.245	0.8 (0.57-1.18)	0.303
**Epidural hematoma**	1.0 (0.70-1.44)	1.000	1.4 (0.95-2.00)	0.104
**Subdural hematoma**	0.6 (0.48-0.87)	0.004	0.6 (0.41-0.85)	0.004
**Subarachnoid hemorrhage**	0.4 (0.29-0.59)	<0.001	0.5 (0.33-0.73)	<0.001
**Intracerebral hematoma**	0.3 (0.12-0.70)	0.005	0.8 (0.43-1.54)	0.562
**Cerebral contusion**	0.6 (0.36-0.89)	0.012	0.3 (0.13-0.58)	<0.001
**Cervical vertebral fracture**	0.1 (0.01-0.70)	0.004	0.1 (0.02-0.98)	0.017
**Maxillofacial trauma, n (%)**				
**Orbital fracture**	0.2 (0.07-0.50)	<0.001	0.4 (0.18-0.88)	0.021
**Nasal fracture**	0.2 (0.04-0.68)	0.005	0.4 (0.11-1.11)	0.070
**Maxillary fracture**	0.1 (0.03-0.19)	<0.001	0.2 (0.10-0.39)	<0.001
**Mandibular fracture**	0.2 (0.06-0.46)	<0.001	0.5 (0.28-1.05)	0.071
**Thoracic trauma, n (%)**				
**Rib fracture**	0.02 (0.01-0.83)	<0.001	0.03 (0.01-0.12)	<0.001
**Sternal fracture**	-	0.399	-	0.625
**Hemothorax**	-	<0.001	-	0.002
**Pneumothorax**	0.2 (0.09-0.63)	0.002	0.2 (0.04-0.66)	0.004
**Hemopneumothorax**	0.2 (0.04-0.62)	0.003	0.1 (0.02-0.77)	0.009
**Lung contusion**	0.6 (0.32-1.32)	0.240	-	0.005
**Thoracic vertebral fracture**	-	0.003	-	0.012
**Abdominal trauma, n (%)**				
**Intra-abdominal injury**	1.0 (0.58-1.64)	1.000	0.7 (0.36-1.47)	0.438
**Hepatic injury**	0.9 (0.54-1.44)	0.640	0.3 (0.11-0.77)	0.011
**Splenic injury**	0.8 (0.40-1.65)	0.625	1.0 (0.46-2.12)	1.000

**Retroperitoneal injury**	-	0.172	-	0.266
**Renal injury**	0.4 (0.10-1.60)	0.243	1.4 (0.56-3.41)	0.590
**Urinary bladder injury**	1.5 (0.35-6.33)	0.646	-	0.625
**Lumbar vertebral fracture**	-	<0.001	-	0.001
**Sacral vertebral fracture**	-	0.018	-	0.042
**Extremity trauma, n (%)**				
**Scapular fracture**	0.2 (0.07-0.51)	<0.001	-	<0.001
**Clavicle fracture**	0.1 (0.04-0.16)	<0.001	0.1 (0.05-0.23)	<0.001
**Humeral fracture**	4.4 (3.70-5.25)	<0.001	4.1 (3.35-5.07)	<0.001
**Radial fracture**	0.4 (0.30-0.55)	<0.001	3.2 (2.68-3.81)	<0.001
**Ulnar fracture**	0.7 (0.47-0.96)	0.032	3.2 (2.59-4.05)	<0.001
**Metacarpal fracture**	0.1 (0.05-0.37)	<0.001	0.1 (0.05-0.45)	<0.001
**Pelvic fracture**	0.2 (0.09-0.47)	<0.001	0.1 (0.02-0.40)	<0.001
**Femoral fracture**	0.5 (0.36-0.68)	<0.001	0.6 (0.42-0.85)	0.003
**Patella fracture**	-	<0.001	0.1 (0.01-0.38)	<0.001
**Tibial fracture**	0.3 (0.19-0.50)	<0.001	0.6 (0.37-0.87)	0.010
**Fibular fracture**	0.5 (0.30-0.75)	0.001	0.5 (0.29-0.84)	0.010
**Calcaneal fracture**	-	<0.001	0.1 (0.06-0.32)	<0.001
**Metatarsal fracture**	0.1 (0.02-0.25)	<0.001	0.2 (0.01-0.53)	<0.001
